# Evaluation of Lingual Tactile Sensitivity Using a Pneumatic Multi‐Point Stimulation Device

**DOI:** 10.1111/joor.70280

**Published:** 2026-07-28

**Authors:** Haruto Kadowaki, Makoto Sasaki, Yasushi Tamada, Yoshihiro Tanaka

**Affiliations:** ^1^ Graduate School of Arts and Sciences Iwate University Morioka Japan; ^2^ Gerodontology, Graduate School of Dental Medicine Hokkaido University Sapporo Japan; ^3^ Graduate School of Engineering Nagoya Institute of Technology Nagoya Japan

**Keywords:** passive and active touch, sensory function, tactile sensitivity, tongue

## Abstract

**Background:**

The sensory function of the tongue is critical for mastication and swallowing. However, its assessment methods remain insufficiently established.

**Objectives:**

In this study, we developed a pneumatic multi‐point stimulation system capable of controlling the location, timing, and pressure of small balloon inflations to simulate the movement and elasticity of food within the oral cavity. Using this system, we established a new method to quantify lingual tactile sensitivity, specifically the ability to detect stimulus point movement under passive touch and pressure differences under active touch.

**Methods:**

We evaluated the age‐related differences in lingual tactile sensitivity in 16 young men (mean age ± standard deviation, 21.9 ± 1.2 years) and 20 older men (74.1 ± 4.1 years) with no history of tongue or related disorders.

**Results:**

The results demonstrated significant age‐related declines in lingual tactile sensitivity under both passive and active touch conditions. Furthermore, these measures showed no significant correlations with conventional sensory tests, including the Semmes–Weinstein monofilament test and the two‐point discrimination test. This suggests that the proposed method captures aspects of lingual sensory function not assessed by existing approaches.

**Conclusion:**

By enabling the quantitative evaluation, using a single device, of not only dynamic sensory perception during passive touch but also active touch, which involves the integration of sensory input (superficial and deep sensation) and motor output, this method has the potential to serve as a novel tool for screening oral hypofunction in older adults and for supporting the assessment of dysphagia, including masticatory dysfunction.

## Graphical abstract

A pneumatic multi‐point stimulation system was developed to quantify lingual sensory function by simulating the movement and elasticity of food within the oral cavity. The system revealed significant age‐related declines in tactile sensitivity for both detection of stimulus point movement during passive touch and discrimination of stimulus point pressure during active touch. This method may serve as a novel tool for screening oral hypofunction in older adults and for supporting the diagnosis of dysphagia, including masticatory dysfunction.

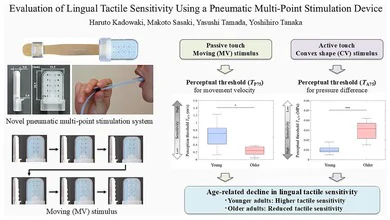

## 1 Introduction

Differences in intraoral sensory information, including the amount, shape, and physical properties of food, influence not only tongue movements during mastication [1, 2] and swallowing [3, 4, 5, 6] but also the movements of other swallowing‐related structures, such as the hyoid bone [7, 8, 9, 10, 11]. Lingual sensory function plays an essential role in feeding and swallowing, including the perception of food properties, bolus formation, and propulsion of the bolus into the pharynx. Consequently, reduced sensory function may delay the triggering of the swallowing reflex and increase the risk of pre‐swallow aspiration [12].

In addition, reduced sensory function of the oral and pharyngeal mucosa is considered one of the causes of food residue, with reduced pharyngeal mucosal sensation particularly contributing to silent aspiration [13, 14]. Food residue increases the risk of aspiration and aspiration pneumonia [15, 16], highlighting the critical role of sensory function in the safe execution of feeding and swallowing.

Aging reduces oral function, swallowing function, and swallowing reserve capacity [17, 18, 19, 20]. Similar age‐related declines are seen in the sensory functions of the tongue, including tactile thresholds, two‐point discrimination thresholds, and stereognostic ability [21, 22, 23, 24, 25]. The Japanese Society of Gerodontology has proposed the concept of oral hypofunction as a preclinical stage preceding dysphagia and masticatory dysfunction [19]. However, standardized clinical methods for assessing oral sensory function have not yet been established, and current diagnostic criteria for oral hypofunction do not include sensory assessment.

Previous studies evaluating oral sensory function have often used the Semmes–Weinstein monofilament test (SWMT) to assess tactile thresholds and the two‐point discrimination test (TPDT) to measure spatial discrimination thresholds [23, 25, 26, 27]. However, the results of these tests can vary depending on the location and force with which the instrument is applied, making them susceptible to examiner‐dependent variability. Moreover, the thresholds obtained using SWMT and TPDT have shown no correlation with the ability to discriminate the hardness or elasticity of gel foods or the viscosity of syrups [28, 29]. Therefore, assessments based on static stimuli applied to a stationary tongue (passive touch) may not adequately reflect sensory function under conditions where the location and intensity of stimuli dynamically change, as occurs during contact between the tongue and the bolus during mastication and swallowing.

When humans perceive food with the tongue, they actively explore the stimulus by moving the tongue, a process referred to as active touch [30, 31, 32, 33]. Active touch is achieved through sensorimotor integration that combines sensory inputs (superficial and deep sensations) with motor outputs. Thus, evaluations based solely on passive touch cannot fully capture lingual sensory function. One approach that considers active touch is the oral stereognosis test, in which test pieces of various shapes are inserted into the oral cavity and participants identify their shapes [21, 24, 27]. However, the physical properties of these test pieces differ substantially from those of real foods with varying hardness and elasticity. Furthermore, this method evaluates integrated sensory input from multiple oral structures, including the lips, teeth, and buccal mucosa, rather than the tongue alone. It also requires comparison between stored representations of shapes in memory and sensory information perceived in the oral cavity, making the results susceptible to influences from cognition and memory. In addition, variations in stimulus shape, material, size, and presentation time across research groups make comparison and standardization difficult.

Given the limitations of existing methods, a simple and quantitative method that considers bolus movement and differences in physical properties is needed to evaluate lingual sensory function. Therefore, the present study proposes a novel method for assessing lingual sensory function using a pneumatic multi‐point stimulation system capable of controlling the location, timing, and pressure of inflation of small balloons. Using this system, lingual tactile sensitivity was quantified from two perspectives: detection of moving stimulus points during passive touch and detection of stimulus pressure during active touch. By comparing lingual tactile sensitivity between younger and older adults, this study also examined whether age‐related changes in lingual sensory function can be detected using the proposed method.

## 2 Methods

### 2.1 Participants

This study included 16 young men aged 20–25 years (mean ± standard deviation, 21.9 ± 1.2 years) and 20 older men aged 68–82 years (74.1 ± 4.1 years) with no history of tongue‐related diseases or conditions. Prior to enrollment, all participants were administered the Mini‐Mental State Examination [34] to confirm the absence of suspected dementia. The study was approved by the ethical review board of our institution and was conducted in accordance with the principles of the Declaration of Helsinki and its later amendments. Before starting the procedures, we explained the study objectives, experimental protocol, and risks to each participant and obtained written consent.

### 2.2 Equipment

A pneumatic multi‐point stimulation system, consisting of a tongue stimulation device and a balloon control unit, was developed to evaluate lingual sensory function. The tongue stimulation device was designed as a multi‐point balloon array and fabricated from medical‐grade silicone. It comprises 10 circular membrane elements (diameter, 3 mm; thickness, 0.2 mm) arranged at 7‐mm intervals both longitudinally and transversely, which expand upon pressurization (Figure 1). The device was mounted in a case made from a biocompatible, autoclavable resin used for surgical guides. A stainless steel tongue depressor was integrated into the case, allowing participants to press the device against their tongue during sensory assessment. In addition, a 5‐mm‐high protrusion was incorporated into the case to serve as a positional reference for the tongue tip during passive touch conditions.

**FIGURE 1:**
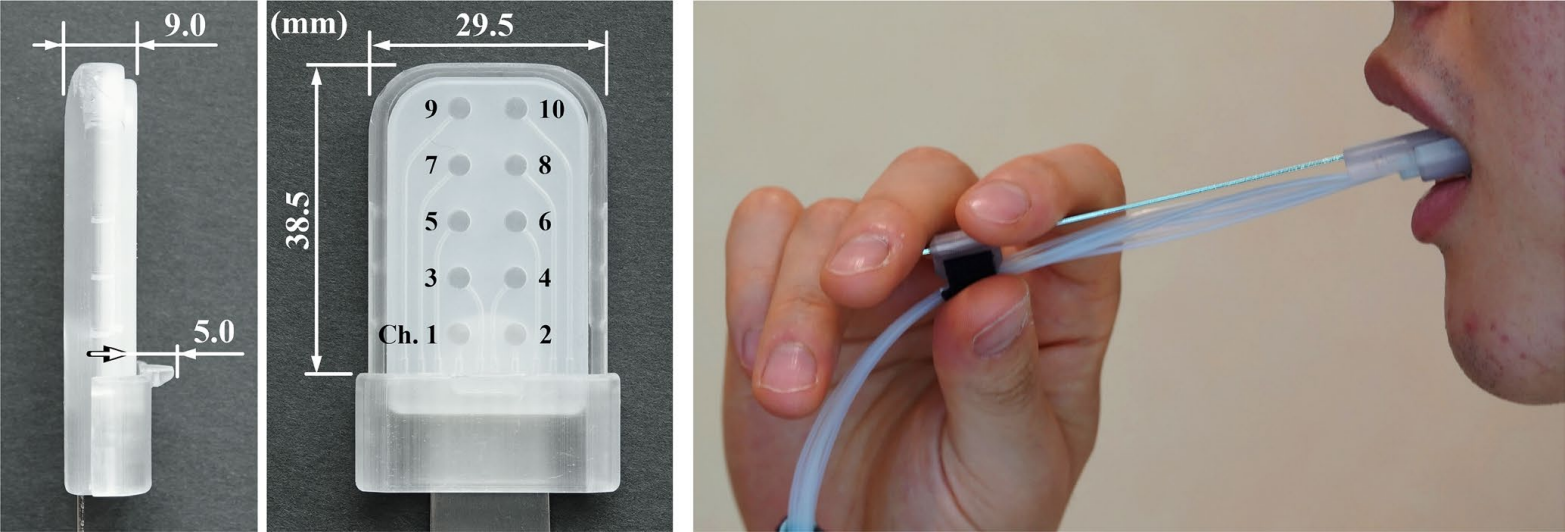
Pneumatic multi‐point stimulation system with ten balloons.

Balloon inflation and deflation were controlled using compressed air from an oil‐free compressor (Seifu Mini, Kyodensha Co. Ltd.). The air was purified using a filter regulator equipped with a 5‐μm filter element (MS2‐LFR‐QS6‐D6‐AR‐MPA‐C‐M‐B, Festo K.K.) and delivered to the tongue stimulation device via piezoelectric valves (VEAB‐L26‐D7‐Q4‐V1‐1R1, Festo K.K.) and nylon tubing. The inflation pressure of each balloon was regulated within a range of 0.0005–0.1000 MPa by applying voltage commands from a D/A converter (NI 9264, National Instruments Corp.), connected to a control PC via USB, to each of the 10 piezoelectric valves. A control program was developed in MATLAB to independently regulate the pressure and timing of inflation and deflation for each balloon. To reduce operational noise, silencers (ANA1‐01, SMC Corporation) were attached to the piezoelectric valves.

### 2.3 Assessment of Oral Function

Among the seven items used to evaluate oral hypofunction by the Japanese Society of Gerodontology [19], six tests were conducted. We did not assess decreased occlusal force because it was considered to have limited relevance to lingual sensory function. Oral hygiene status was evaluated by measuring the number of microorganisms on the dorsal surface of the tongue using an oral bacterial counter (NP‐BCM01‐A, Panasonic Corporation). Oral dryness was assessed by measuring mucosal moisture at the central dorsum of the tongue using an oral moisture meter (Mucus, Life Co. Ltd.). Tongue–lip motor function was determined using oral diadochokinesis, with the number of repetitions of the syllables /pa/, /ta/, and /ka/ over 5 s measured using an automatic counter (Kenko‐kun, Takei Scientific Instruments Co. Ltd.). Maximum tongue pressure was measured using a tongue pressure measurement device (TPM‐02E, JMS Co. Ltd.). Masticatory function was assessed by measuring the amount of glucose eluted from gummy jelly using a glucose sensor (GS‐II, GC Japan). Swallowing function was evaluated using the Eating Assessment Tool (EAT‐10) [35].

For comparison with conventional methods, tactile thresholds were assessed using SWMT with five filaments (SOT‐DM10A, Sakai Medical Co. Ltd.; #1.65, #2.36, #2.44, #2.83, and #3.22, corresponding to target forces of 0.008, 0.02, 0.04, 0.07, and 0.16 gf, respectively). Two‐point discrimination thresholds were measured using TPDT with distances ranging from 0.5 to 7.0 mm in 0.5‐mm increments. The test site was the central dorsum of the tongue (12 mm posterior to the tongue tip). During testing, participants wore an eye mask, and thresholds were determined using the method of limits.

### 2.4 Data Acquisition Under Passive Touch

Participants were seated and held a tongue depressor fitted with the tongue stimulation device. They gently pressed the device against the tongue while positioning the tongue tip at the protrusion shown in Figure 1. To eliminate auditory cues from the piezoelectric valves, participants wore noise‐cancelling headphones (WH‐1000XM5, Sony Corporation) playing white noise. Participants were presented with two stimulus patterns separated by a 3‐s interstimulus interval and were asked to indicate, using a two‐alternative forced‐choice (2AFC) paradigm, which pattern involved perceived movement of the stimulus point (Figure 2).

**FIGURE 2:**
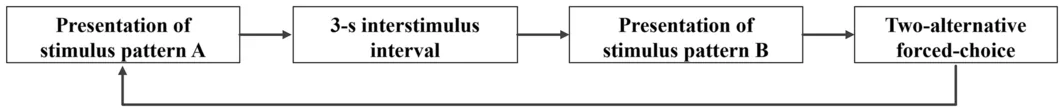
Two‐alternative forced‐choice (2AFC) paradigm.

Two types of stimuli were used: a reference (Ref) stimulus, in which all balloons were inflated simultaneously, and a moving (MV) stimulus, in which the balloons were sequentially inflated to create the perception of movement from the anterior to the posterior tongue (Figure 3). To maintain bilateral symmetry, adjacent channels (Ch. 1–2, 3–4, 5–6, 7–8, and 9–10) were actuated synchronously. The inflation pressure was set to 0.1 MPa (corresponding to ~1 mm of expansion). A total of 11 MV stimuli were tested, with the temporal delay (*t*) and corresponding stimulus velocity (*v*) shown in Table 1. The presentation duration of both the Ref stimulus and the MV stimulus was set to 1.14 s. Each stimulus was presented to the participants twice consecutively, with a 1‐s interstimulus interval (Figure 3). When the Ref stimulus and the MV stimulus were each assigned to either stimulation pattern A or stimulation pattern B shown in Figure 2, a total of 22 possible stimulus combinations could be presented to the participants. In this experiment, these combinations were presented five times each in a randomized order, resulting in a 2AFC task comprising a total of 110 trials. During the experiment, participants were instructed not to move their tongues. Rest and water intake were allowed between trials.

**FIGURE 3:**
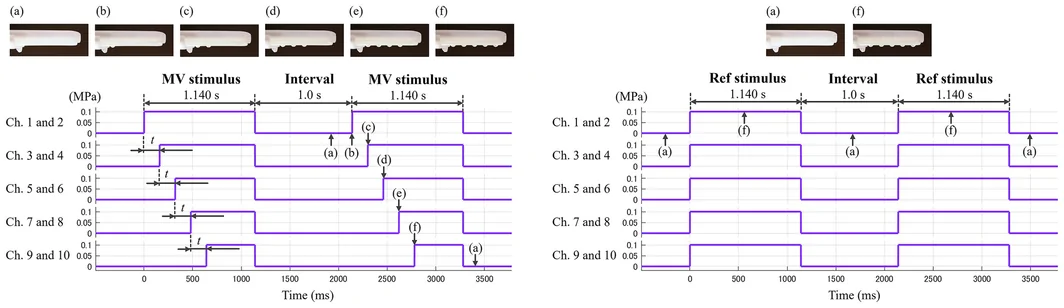
MV stimulus and Ref stimulus.

**TABLE 1:** Movement velocities of stimulus points in MV stimuli.

Stimulus condition	1	2	3	4	5	6	7	8	9	10	11
Time delay *t*, s	0.005	0.007	0.010	0.014	0.020	0.028	0.040	0.057	0.080	0.113	0.160
Moving velocity *v*, m/s	1.400	1.000	0.700	0.500	0.350	0.250	0.175	0.123	0.088	0.062	0.044

### 2.5 Data Acquisition Under Active Touch

Participants were seated and held the device as in the passive condition while wearing noise‐cancelling headphones with white noise. In this condition, only six balloons (Ch. 1–6) were used. Channels were paired symmetrically (Ch. 1–2, 3–4, and 5–6). Using a 2AFC paradigm (Figure 2), participants were asked to identify which stimulus pattern produced a stronger expansion at the central balloons (Ch. 3–4) compared to the surrounding balloons (Ch. 1–2 and Ch. 5–6).

Two types of stimuli were used: a reference stimulus (Ref), in which all six balloons were inflated at 0.05 MPa (~0.6 mm of expansion), and a comparison stimulus, in which the central balloons had higher pressure, forming a convex shape (CV stimulus) (Figure 4). A total of 11 CV stimuli were tested, with the pressure differences (*pd*) shown in Table 2. Each stimulus was presented for 5 s, during which participants were allowed to actively explore the stimulus by moving their tongue. There were a total of 22 possible combinations in which the Ref stimulus and the CV stimulus were assigned to either stimulation pattern A or stimulation pattern B shown in Figure 2. These combinations were presented five times each in a randomized order, resulting in a 2AFC task consisting of 110 trials. Rest and water intake were allowed between trials.

**FIGURE 4:**
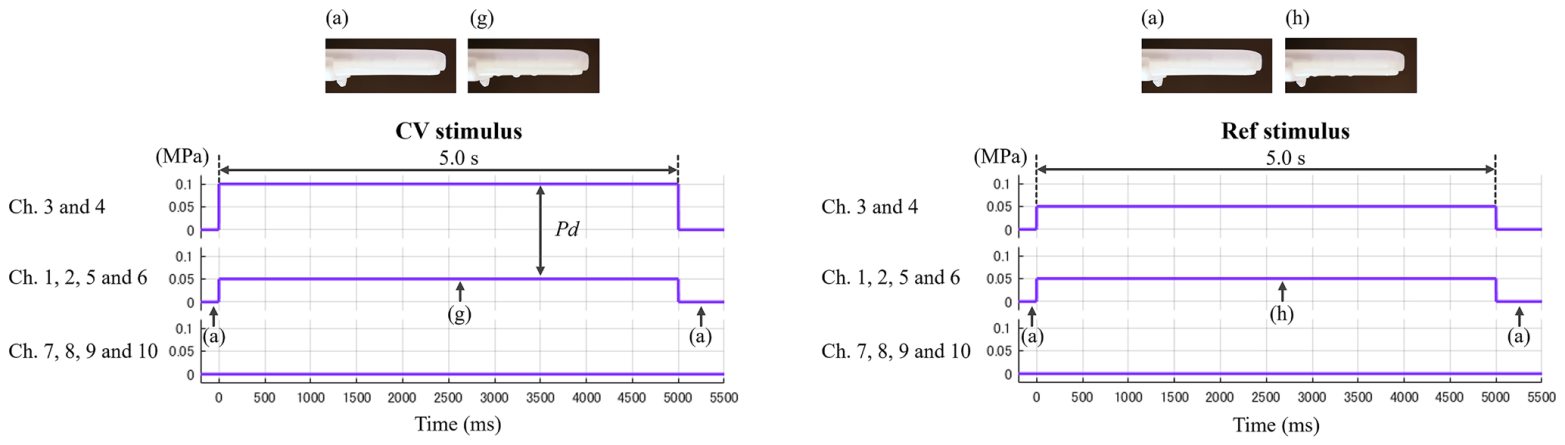
CV stimulus and Ref stimulus.

**TABLE 2:** Pressure differences of stimulus points in CV stimuli.

Stimulus condition	1	2	3	4	5	6	7	8	9	10	11
Pressures of Ch.3 and 4, MPa	0.051	0.055	0.060	0.065	0.070	0.075	0.080	0.085	0.090	0.095	0.100
Pressure difference *pd*, MPa	0.001	0.005	0.010	0.015	0.020	0.025	0.030	0.035	0.040	0.045	0.050

### 2.6 Estimation of Perceptual Thresholds

In the 2AFC paradigm [36, 37], the correct response rate approaches 100% as the difference between the reference and comparison stimuli increases and approaches 50% (chance level) as the difference decreases (Figure 5). In this study, psychometric functions were fitted using a cumulative normal distribution within the range of 50%–100%, and the stimulus intensity corresponding to 75% correct responses was defined as the tactile threshold:

(1) $$f\left(z\right)=0.75+0.25\mathrm{erf}\left(\frac{z-z_{0}}{\sqrt{2}s}\right)$$

(2) $$\mathrm{erf}\left(x\right)=\frac{2}{\sqrt{\pi }}\int_{-x}^{x}e^{-t^{2}}\mathrm{dt}$$

where *z* represents stimulus intensity, *z*_0_ the mean, and *s* the standard deviation.

**FIGURE 5:**
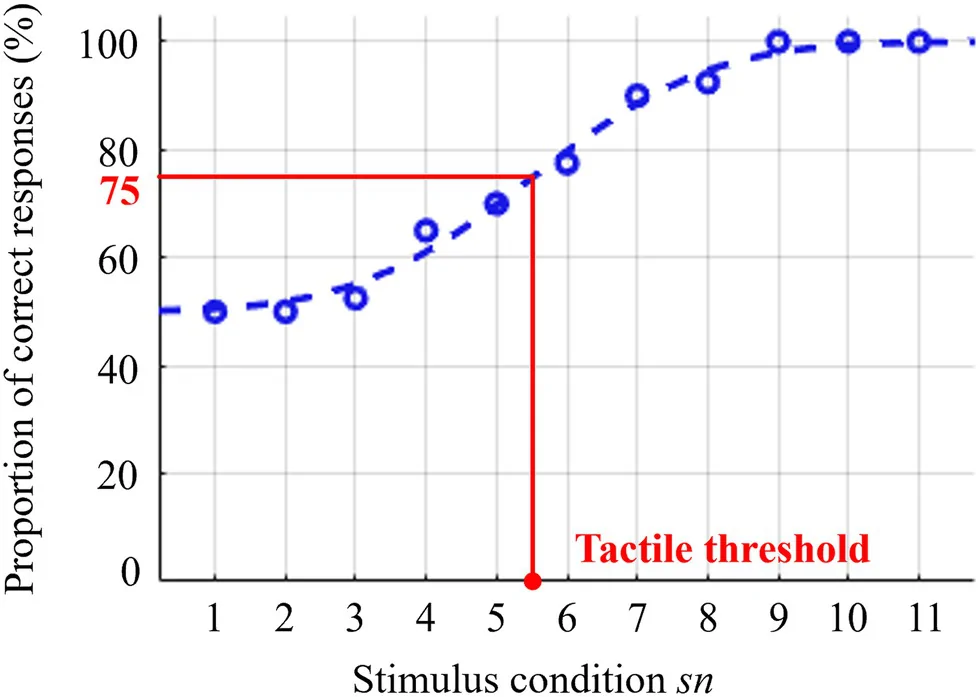
Definition of lingual tactile sensitivity using the 2AFC task.

For MV stimuli, the stimulus condition (*sn*) corresponds to the velocity (*v*), and the passive‐touch threshold (*T*_P75_) indicates the maximum detectable movement velocity; higher values reflect higher tactile sensitivity. For CV stimuli, the stimulus condition *sn* corresponds to the pressure difference (*pd*), and the active‐touch threshold (*T*_A75_) indicates the minimum detectable pressure difference; higher values reflect lower tactile sensitivity. Data for which *T*_P75_ or *T*_A75_ exceeded the range of stimulus conditions presented to the participants were excluded from the analysis as outliers, as the reliability of such results could not be ensured. Outliers can be classified into two types: those in which the perceptual threshold exceeds the upper limit of the measurable range, and those in which it falls below the lower limit.

### 2.7 Statistical Analysis

Group comparisons between younger and older participants were performed using the Wilcoxon rank‐sum test. Associations between oral function measures and *T*_P75_/*T*_A75_ were analysed using Pearson's correlation coefficients. Statistical analyses were conducted using MATLAB R2024b (The MathWorks Inc.) with the Statistics and Machine Learning Toolbox.

## 3 Results

### 3.1 Comparison of Oral Function Between Young and Older Participants

A comparison of oral function between the younger and older groups is shown in Table 3. Compared with the younger group, the older group exhibited significant reductions in oral hygiene status, tongue–lip motor function, and tongue pressure. In contrast, no significant differences were observed between the groups in SWMT or TPDT at the central dorsum of the tongue.

**TABLE 3:** Oral function data in the young and older groups.

Oral function	Threshold [19, 38]	Young group	Older group	*p*
Assessment items for oral hypofunction				
Oral hygiene, ×10^6^ cfu/mL	≥ 3.162	1.9 ± 1.8	8.8 ± 7.6	< 0.001
Oral dryness, arb. unit	< 27.0	26.7 ± 3.5	28.0 ± 1.5	n.s.
Tongue–lip motor function, times/s				
/pa/	< 6	6.9 ± 0.6	6.2 ± 0.9	< 0.05
/ta/	< 6	7.5 ± 0.8	6.2 ± 0.7	< 0.001
/ka/	< 6	7.0 ± 0.8	5.6 ± 0.8	< 0.001
Tongue pressure, kPa	< 30	47.8 ± 9.0	30.7 ± 8.5	< 0.001
Masticatory function, mg/dL	< 100	186.8 ± 35.0	186.9 ± 59.4	n.s.
Swallowing function score	≥ 3	0.4 ± 0.6	1.5 ± 2.7	n.s.
Assessment items for oral sensory function				
SWMT, ×10^−2^ gf	—	2.7 ± 1.7	3.4 ± 2.0	n.s.
TPDT, mm	—	1.5 ± 0.7	1.7 ± 1.1	n.s.

Abbreviations: n.s., not significant; SWMT, Semmes–Weinstein monofilament test; TPDT, two‐point discrimination test.

### 3.2 Lingual Sensory Function Under Passive Touch

By excluding participants whose *T*_P75_ for MV stimuli fell outside the stimulus range (velocity, 0.044–1.400 m/s), 9 of the 16 younger participants and 12 of the 20 older participants were included in the analysis. *T*_P75_ represents the maximum velocity at which movement of the stimulus point toward the posterior tongue can be perceived. *T*_P75_ was significantly lower in the older group than in the younger group (Figure 6). The median (IQR) *T*_P75_ was 0.71 (0.42–0.85) m/s in the younger group but 0.24 (0.14–0.35) m/s in the older group.

**FIGURE 6:**
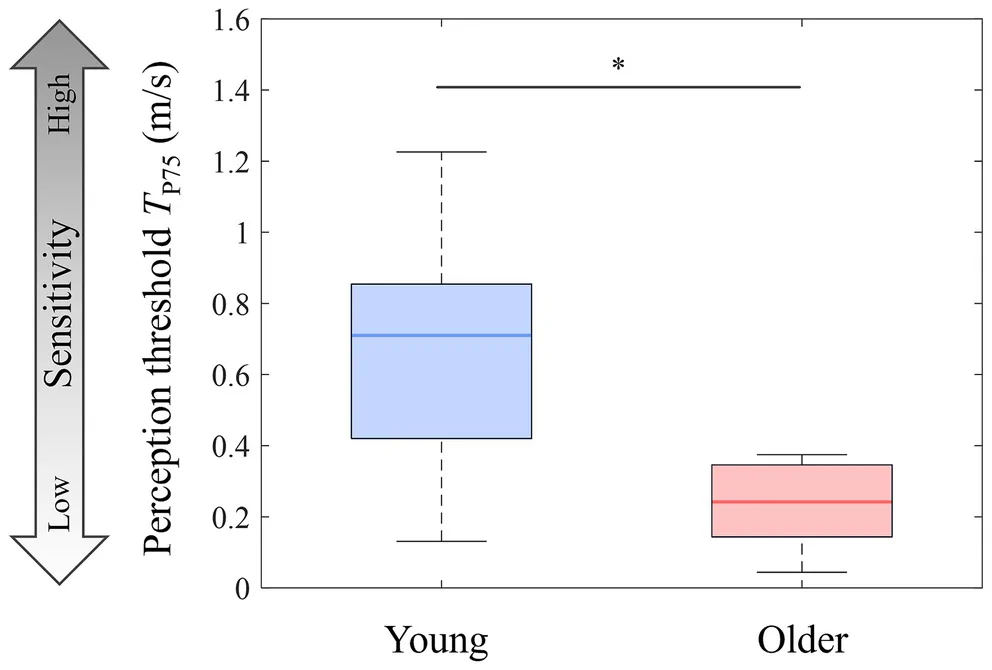
Passive‐touch threshold *T*_P75_ for MV stimuli. *p < 0.05.

Among the oral function measures, only age (*r* = 0.54, *p* < 0.05) and oral diadochokinesis /ta/ (*r* = 0.64, *p* < 0.01) showed significant correlations with *T*_P75_. These results indicate that younger individuals and those with higher /ta/ repetition rates were able to perceive faster stimulus movement, reflecting higher tactile sensitivity of the tongue.

In the MV stimulus experiment, among participants whose *T*_P75_ values fell outside the measurable range, six younger participants and one older participant exceeded the upper sensitivity limit, whereas one younger participant and seven older participants fell below the lower sensitivity limit. A comparison of older participants whose *T*_P75_ values fell within the measurable range (*n* = 12; included in the analysis) with those whose values fell below the lower sensitivity limit (*n* = 7; excluded from the analysis) revealed significant differences in age (72.8 ± 3.5 vs. 77.1 ± 3.1 years, *p* < 0.05), oral diadochokinesis for /pa/ (6.5 ± 0.8 vs. 5.6 ± 0.8, *p* < 0.05), and oral diadochokinesis for /ta/ (6.5 ± 0.3 vs. 5.6 ± 0.6, *p* < 0.01).

### 3.3 Lingual Sensory Function Under Active Touch

Only participants whose *T*_A75_ for CV stimuli fell within the stimulus range (pressure difference, 0.001–0.050 MPa) were included in the analysis: 14 of the 16 younger participants and 12 of the 20 older participants. *T*_A75_ represents the minimum pressure difference required to discriminate between balloons using the tongue and was significantly higher in the older than younger groups (Figure 7). The median (IQR) *T*_A75_ was 0.009 (0.008–0.012) MPa in the younger group and 0.032 (0.021–0.037) MPa in the older group, corresponding to expansion height differences of 0.08 (0.07–0.11) mm and 0.28 (0.18–0.32) mm, respectively.

**FIGURE 7:**
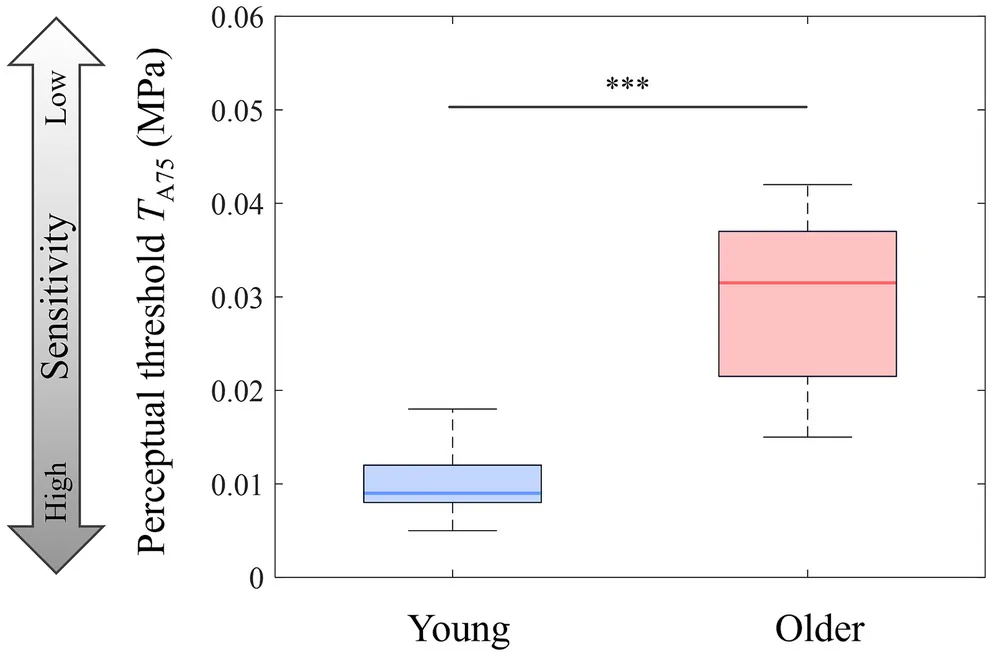
Active‐touch threshold *T*_A75_ for CV stimuli. ***p < 0.001.

Among the oral function measures, significant correlations with *T*_A75_ were found for age (*r* = 0.76, *p* < 0.001), tongue pressure (*r* = −0.43, *p* < 0.001), and oral diadochokinesis /ta/ (*r* = −0.55, *p* < 0.05). These findings indicate that younger individuals, those with greater maximum tongue pressure, and those with higher /ta/ repetition rates were able to discriminate smaller pressure differences, indicating higher lingual tactile sensitivity.

In the CV stimulus experiment, among participants whose *T*_A75_ values fell outside the measurable range, one younger participant exceeded the upper sensitivity limit, one younger participant fell below the lower sensitivity limit, and eight older participants fell below the lower sensitivity limit. When comparing older participants whose *T*_A75_ values fell within the measurable range (*n* = 12; included in the analysis) with those whose values fell below the lower sensitivity limit (*n* = 8; excluded from the analysis), a significant difference was observed only in swallowing function, as assessed by the EAT‐10 (0.58 ± 1.66 vs. 2.88 ± 3.33, *p* < 0.05).

### 3.4 Relationship Between Passive and Active Touch

Across both experiments, 8 younger participants and 7 older participants had both *T*_P75_ and *T*_A75_ values within the stimulus range. The distribution of *T*_P75_ and *T*_A75_ suggested that younger participants tended to cluster in the lower‐right region of the plot (indicating higher sensitivity for both stimuli), whereas older participants tended to cluster in the upper‐left region (indicating lower sensitivity). However, no significant correlation was observed between *T*_P75_ and *T*_A75_ (Figure 8).

**FIGURE 8:**
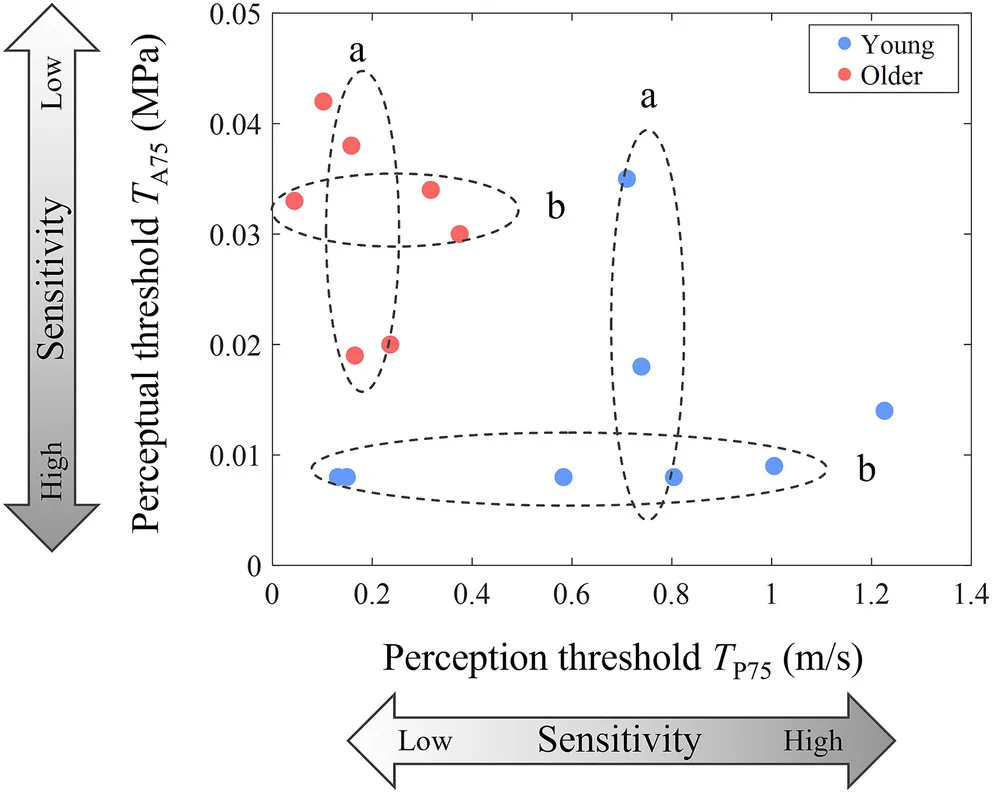
Relationship between *T*_P75_ and *T*_A75._ Participants enclosed by the dashed ellipse (a) exhibited comparable *T*_P75_ values but differing distributions of *T*_A75_. In contrast, participants enclosed by the dashed ellipse (b) exhibited comparable *T*_A75_ values but differing distributions of *T*_P75_.

## 4 Discussion

### 4.1 Lingual Tactile Sensitivity to MV Stimuli

In the present study, *T*_P75_ reflects lingual sensory function during passive touch. *T*_P75_ was significantly lower in the older group than in the younger group, and similar trends were observed among participants excluded from the analysis. This indicates that the older adults required slower velocities to detect the movement of the stimulus point and thereby exhibited reduced lingual tactile sensitivity. Previous ultrasonographic studies have reported that intraoral bolus transport velocity during swallowing ranges from approximately 0.22 to 0.59 m/s in healthy young adults [39]. Therefore, older individuals with reduced sensitivity to MV stimuli may have difficulty accurately perceiving rapid positional changes of the bolus as it moves along the tongue surface during swallowing.

No significant correlations were observed between the *T*_P75_ and conventional tactile measures such as the SWMT and TPDT. This lack of correlation may be due to the involvement of different mechanoreceptive units. In the human lingual nerve, four types of units have been reported [40, 41]: rapidly adapting (RA) units; two types of slowly adapting units (SAI and SAII) resembling the fast‐ and slowly adapting type I and type II afferents, respectively, of the glabrous skin of the human hand; and deep units that encode information about tongue movements. While the SWMT and TPDT primarily reflect the responses of SAI units to sustained pressure, MV stimuli likely engage RA units that respond to dynamic stimuli. Thus, the proposed method may capture a distinct aspect of lingual sensory function that is not assessed by conventional tests.

### 4.2 Lingual Tactile Sensitivity to CV Stimuli

*T*_A75_ represents sensory function during active touch. *T*_A75_ was significantly higher in the older group than in the younger group, and similar trends were observed among participants excluded from the analysis. This indicates that older adults required larger pressure differences to discriminate stimuli, reflecting reduced lingual tactile sensitivity. During feeding and swallowing, the ability to actively press and identify intraoral contents is essential for bolus formation and transport. Therefore, decreased sensitivity to CV stimuli in older adults may adversely affect these processes.

Similar to *T*_P75_, *T*_A75_ showed no significant correlations with SWMT or TPDT. Active touch requires the real‐time integration of cutaneous (RA, SAI, SAII) and proprioceptive inputs with motor control. The absence of a correlation with SWMT and TPDT, which primarily assess superficial sensation, is therefore reasonable. Previous studies have also reported that SWMT and TPDT are not significantly associated with the ability to discriminate food texture properties such as hardness, elasticity, or viscosity [28, 29], suggesting limitations of these tests in clarifying the relationship of oral sensory function with mastication and swallowing function.

### 4.3 Relationship Between Passive and Active Touch

No significant correlation was found between *T*_P75_ and *T*_A75_ (Figure 8). However, the distribution patterns suggested a dissociation between passive and active sensory abilities: even when *T*_P75_ values were comparable, the distribution of *T*_A75_ varied (Figure 8a) and, similarly, variability in *T*_P75_ was observed among participants with comparable *T*_A75_ values (Figure 8b). Active touch relies on sensorimotor integration, combining sensory input (superficial and deep sensation) with motor output. Therefore, inter‐individual variability in motor function may partly explain the observed dissociation. Supporting this interpretation, *T*_A75_ was significantly correlated with maximum tongue pressure and oral diadochokinesis (/ta/), indicating that individuals with better lingual motor function tended to exhibit higher perceptual sensitivity during active touch. However, because /ta/ was also correlated with *T*_P75_, further studies with larger sample sizes are needed to clarify the relationship between passive and active sensory functions and to better characterize individual sensory profiles.

### 4.4 Age‐Related Changes in Lingual Sensory Function

Age‐related declines in lingual sensory function were clearly demonstrated not only in the group comparisons shown in Figures 6 and 7 but also in the distributions of *T*_P75_ and *T*_A75_. These changes may be attributed to physiological alterations in the lingual mucosal tissues, as well as to functional declines in both the peripheral and central nervous systems [42, 43, 44, 45, 46, 47]. A previous study that evaluated oral texture perception using an aqueous suspension containing crystalline cellulose reported that individuals with high sensitivity to roughness exhibited significantly greater tongue surface roughness [42]. In addition, studies investigating the three‐dimensional structure of the tongue have reported that aging is associated with the loss of protrusions of the filiform papillae and flattening of the epithelium [43], that older adults with dysphagia exhibit reduced tongue surface roughness compared with those without dysphagia [44], and that higher fungiform papillae density is associated with greater perceptual sensitivity in letter identification tasks [45]. Furthermore, immunohistochemical studies have demonstrated that filiform papillae contain a high density of end bulbs of Krause, subepithelial neuronal elements, and NFH‐positive neurons innervating the epithelium, whereas fungiform papillae contain Meissner‐like corpuscles and dense NFH‐positive intraepithelial neurons surrounding taste buds [48]. These findings strongly suggest that the morphology and density of filiform and fungiform papillae play an important role in lingual sensory function. On the other hand, studies using artificial fingertips have suggested that fingerprints contribute to spectral selection and amplification of tactile signals [49], and it is conceivable that tongue surface roughness itself may serve a function analogous to that of fingerprints. Although the present study did not directly measure the morphology or density of filiform and fungiform papillae or examine their relationships with *T*_P75_ and *T*_A75_, it is reasonable to assume that age‐related changes in the three‐dimensional structure of the tongue, similar to those reported in previous studies, were also present in the older participants in this study.

### 4.5 Limitations and Future Directions

In this study, we developed a novel method for evaluating lingual sensory function in response to both MV and CV stimuli using a single device. The stimuli employed in this study focused on the movement velocity of stimulus points and differences in pressure generated by small balloons, which were designed to simulate bolus movement and variations in physical properties during mastication and swallowing. The experimental results demonstrated that the proposed method could detect age‐related declines in sensory function, as reflected by both tactile thresholds; that is, *T*_A75_ for active touch and *T*_P75_ for passive touch. Neither *T*_A75_ nor *T*_P75_ showed significant correlations with conventional measures such as SWMT and TPDT, suggesting that this method may provide a novel perspective for evaluating lingual sensory function.

However, the *T*_P75_ and *T*_A75_ values in the older group were markedly lower than expected, and older individuals with oral hypofunction tended to fall outside the measurable range. In the MV stimulus experiment, although occlusal force was not assessed among the seven diagnostic items for oral hypofunction [19] in this study, the proportion of older participants who exhibited functional decline in three or more of the six assessed items was 41.7% (5 of 12) in the analysis group and 85.7% (6 of 7) in the non‐analysis group. A similar tendency was observed in the CV stimulus experiment, in which the corresponding proportions were 58.3% (7 of 12) in the analysis group and 75.0% (6 of 8) in the non‐analysis group. These findings suggest that older participants excluded from the analysis tended to be older, exhibited reduced tongue and lip motor function, and were more likely to present with oral hypofunction.

To validate the effectiveness of our pneumatic multi‐point stimulation system for assessing sensory function, we adopted a 2AFC paradigm [37] due to its psychophysical reliability and reduced susceptibility to response bias. Nevertheless, expanding the stimulus presentation range for movement velocity in MV stimuli (Table 1) and pressure differences in CV stimuli (Table 2) in this paradigm would inevitably increase the number of stimulus presentations and responses required, leading to longer evaluation times and increased participant fatigue. Therefore, it was necessary to limit the stimulus range. Accordingly, the predetermined ranges were based on pilot experiments conducted with a small number of healthy young participants. However, the degree of functional decline observed in the older group exceeded expectations. While exceeding the upper sensitivity limit does not pose a major issue for functional evaluation, falling below the lower sensitivity limit represents a limitation that must be addressed to establish criteria (cutoff value) for sensory function decline. Potential improvements include redesigning the balloon structure to increase the contact area with the tongue, thereby enhancing stimulus perception in older individuals, as well as expanding the range of movement velocities and pressure differences presented. Furthermore, to overcome the limitations of the 2AFC paradigm associated with an expanded stimulus range, the adoption of simplified psychophysical methods, such as the method of limits, may enable the efficient measurement of *T*_P75_ and *T*_A75_ within an appropriate range in a shorter time.

It remains unclear how *T*_P75_ and *T*_A75_, as proposed in this study, correspond to actual functional aspects of swallowing, such as bolus formation, propulsion into the pharynx, and the presence or degree of residue. In addition, further investigation is required to clarify their relationships with the presence and severity of dysphagia, underlying pathophysiological characteristics, and sex differences. Therefore, future studies should aim to elucidate the relationships of *T*_P75_ and *T*_A75_ with swallowing function through comparisons with FEES and VFSS. Moreover, to achieve a more comprehensive understanding of swallowing dynamics, future analyses should incorporate not only lingual sensory function but also sensory inputs from other intraoral regions, such as the palate, along with multimodal information, including visual, gustatory, and thermal perception.

## 5 Conclusion

In this study, we developed a pneumatic multi‐point stimulation system to quantify lingual sensory function, which plays a critical role in feeding and swallowing, by simulating the movement and elasticity of food within the oral cavity. A comparison between younger and older adults revealed significant age‐related declines in tactile sensitivity for both detection of stimulus point movement during passive touch and discrimination of stimulus point pressure during active touch. By enabling the quantitative assessment not only of dynamic stimuli during passive touch but also of active touch, which involves the integration of sensory input (superficial and deep sensation) and motor output, this method may serve as a novel tool for screening oral hypofunction in older adults and for supporting the diagnosis of dysphagia, including masticatory dysfunction.

## Author Contributions

**Haruto Kadowaki:** investigation, data curation, formal analysis, writing – original draft, methodology, software, visualization. **Makoto Sasaki:** conceptualization, methodology, validation, formal analysis, supervision, funding acquisition, project administration, resources, writing – review and editing, writing – original draft. **Yasushi Tamada:** validation, writing – review and editing, methodology. **Yoshihiro Tanaka:** methodology, validation, writing – review and editing, formal analysis.

## Funding

This work was supported by the Japan Society for the Promotion of Science (JSPS) KAKENHI (Grant Numbers JP24K22221 and JP25K03503; Principal Investigator: Makoto Sasaki) and by the Soft‐Path Science and Engineering Research Center, Iwate University.

## Ethics Statement

This study was carried out in accordance with the principles embodied in the Declaration of Helsinki and its later amendments and was approved by the Medical Research Ethics Review Board of Iwate University (Approval No. 202317).

## Consent

Participants were informed of the experiment's purpose and procedures, and informed consent was obtained prior to implementation.

## Conflicts of Interest

The authors declare no conflicts of interest.

## Data Availability

The datasets used in the current study are not publicly available due to ethical restrictions and concerns regarding participant privacy. However, data supporting the findings of this study can be obtained from the corresponding author upon reasonable request.

## References

[1] Y. Manda , N. Kodama , N. Maeda , and S. Minagi , “Effect of Food Properties and Chewing Condition on the Electromyographic Activity of the Posterior Tongue,” Journal of Oral Rehabilitation 46 (2019): 511–517, 10.1111/joor.12774. 30724370

[2] T. Iida , K. Matsuo , Y. Iida , et al., “Kinematics of Lateral Tongue‐Pushing Movement in Coordination With Masticatory Jaw Movement: An Anteroposterior Projection Videofluorographic Study,” Physiology & Behavior 270 (2023): 114315, 10.1016/j.physbeh.2023.114315. PMC10530480 37536619

[3] C. L. Lazarus , J. A. Logemann , A. W. Rademaker , et al., “Effects of Bolus Volume, Viscosity, and Repeated Swallows in Nonstroke Subjects and Stroke Patients,” Archives of Physical Medicine and Rehabilitation 74 (1993): 1066–1070, 10.1016/0003-9993(93)90063-g. 8215858

[4] L. Reimers‐Neils , J. Logemann , and C. Larson , “Viscosity Effects on EMG Activity in Normal Swallow,” Dysphagia 9 (1994): 101–106, 10.1007/BF00714596. 8005004

[5] J. L. Miller and K. L. Watkin , “The Influence of Bolus Volume and Viscosity on Anterior Lingual Force During the Oral Stage of Swallowing,” Dysphagia 11 (1996): 117–124, 10.1007/BF00417901. 8721070

[6] G. Chi‐Fishman and B. Sonies , “Effects of Systematic Bolus Viscosity and Volume Changes on Hyoid Movement Kinematics,” Dysphagia 17 (2002): 278–287, 10.1007/s00455-002-0070-7. 12355143

[7] W. J. Dodds , K. M. Man , I. J. Cook , P. J. Kahrilas , E. T. Stewart , and M. K. Kern , “Influence of Bolus Volume on Swallow‐Induced Hyoid Movement in Normal Subjects,” American Journal of Roentgenology 150 (1988): 1307–1309, 10.2214/ajr.150.6.1307. 3259369

[8] I. J. Cook , W. J. Dodds , R. O. Dantas , et al., “Timing of Videofluoroscopic, Manometric Events, and Bolus Transit During the Oral and Pharyngeal Phases of Swallowing,” Dysphagia 4 (1989): 8–15, 10.1007/BF02407397. 2640180

[9] R. O. Dantas , W. J. Dodds , B. T. Massey , and M. K. Kern , “The Effect of High‐ vs Low‐Density Barium Preparations on the Quantitative Features of Swallowing,” American Journal of Roentgenology 153 (1989): 1191–1195, 10.2214/ajr.153.6.1191. 2816631

[10] R. O. Dantas , M. K. Kern , B. T. Massey , et al., “Effect of Swallowed Bolus Variables on Oral and Pharyngeal Phases of Swallowing,” American Journal of Physiology 258 (1990): G675–G681, 10.1152/ajpgi.1990.258.5.G675. 2333995

[11] N. Ueda , K. Nohara , Y. Kotani , N. Tanaka , K. Okuno , and T. Sakai , “Effects of the Bolus Volume on Hyoid Movements in Normal Individuals,” Journal of Oral Rehabilitation 40 (2013): 491–499, 10.1111/joor.12060. 23675892

[12] J. A. Logemann , Evaluation and Treatment of Swallowing Disorders, 2nd ed. (Pro‐Ed, 1998).

[13] B. Labeit , A. Jung , S. Ahring , et al., “Relationship Between Post‐Stroke Dysphagia and Pharyngeal Sensory Impairment,” Neurological Research and Practice 5 (2023): 7, 10.1186/s42466-023-00233-z. PMC9933330 36793109

[14] T. Marian , J. B. Schröder , P. Muhle , et al., “Pharyngolaryngeal Sensory Deficits in Patients With Middle Cerebral Artery Infarction: Lateralization and Relation to Overall Dysphagia Severity,” Cerebrovascular Diseases Extra 7 (2017): 130–139, 10.1159/000479483. PMC5730110 28972945

[15] C. M. Steele , M. Peladeau‐Pigeon , E. Barrett , and T. S. Wolkin , “The Risk of Penetration‐Aspiration Related to Residue in the Pharynx,” American Journal of Speech‐Language Pathology 29 (2020): 1608–1617, 10.1044/2020_AJSLP-20-00042. PMC7893525 32598168

[16] R. R. Santos , A. V. Sales , P. C. Cola , et al., “Association Between Pharyngeal Residue and Posterior Oral Spillage With Penetration and Aspiration in Stroke,” Codas 26 (2014): 231–234, 10.1590/2317-1782/201420140476. 25118920

[17] J. A. Logemann , “Effects of Aging on the Swallowing Mechanism,” Otolaryngologic Clinics of North America 23 (1990): 1045–1056. 2074979

[18] J. A. Logemann , “Critical Factors in the Oral Control Needed for Chewing and Swallowing,” Journal of Texture Studies 45 (2014): 173–179, 10.1111/jtxs.12053. PMC4224116 25386030

[19] S. Minakuchi , K. Tsuga , K. Ikebe , et al., “Oral Hypofunction in the Older Population: Position Paper of the Japanese Society of Gerodontology in 2016,” Gerodontology 35 (2018): 317–324, 10.1111/ger.12347. 29882364

[20] K. Iyota , S. Mizutani , S. Oku , et al., “A Cross‐Sectional Study of Age‐Related Changes in Oral Function in Healthy Japanese Individuals,” International Journal of Environmental Research and Public Health 17 (2020): 1376, 10.3390/ijerph17041376. PMC7068434 32093362

[21] K. H. Calhoun , B. Gibson , L. Hartley , J. Minton , and J. A. Hokanson , “Age‐Related Changes in Oral Sensation,” Laryngoscope 102 (1992): 109–116, 10.1288/00005537-199202000-00001. 1738279

[22] C. H. Smith , J. A. Logemann , W. R. Burghardt , S. G. Zecker , and A. W. Rademaker , “Oral and Oropharyngeal Perceptions of Fluid Viscosity Across the Age Span,” Dysphagia 21 (2006): 209–217, 10.1007/s00455-006-9045-4. 17203333

[23] S. Teranaka , T. Shibaji , S. Minakuchi , and H. Uematsu , “Age‐Related Changes in Oral Mechanosensitivity of Symptom‐Free Subjects,” Journal of Medical and Dental Sciences 55 (2008): 61–69. 19845151

[24] S. Kawagishi , F. Kou , K. Yoshino , T. Tanaka , and S. Masumi , “Decrease in Stereognostic Ability of the Tongue With Age,” Journal of Oral Rehabilitation 36 (2009): 872–879, 10.1111/j.1365-2842.2009.02005.x. 19840359

[25] R. Nishimura , M. Yoshida , H. Shigeishi , et al., “Age‐Related Changes in Oral Tactile and Thermal Sensation Throughout Adulthood,” Odontology 109 (2021): 710–718, 10.1007/s10266-021-00594-4. 33635472

[26] M. W. Heft and M. E. Robinson , “Age Differences in Orofacial Sensory Thresholds,” Journal of Dental Research 89 (2010): 1102–1105, 10.1177/0022034510375287. PMC3318051 20651093

[27] C. A. Boliek , J. M. Rieger , S. Y. Li , Z. Mohamed , J. Kickham , and K. Amundsen , “Establishing a Reliable Protocol to Measure Tongue Sensation,” Journal of Oral Rehabilitation 34 (2007): 433–441, 10.1111/j.1365-2842.2007.01735.x. 17518978

[28] T. Aktar , J. Chen , R. Ettelaie , and M. Holmes , “Tactile Sensitivity and Texture Discrimination,” Journal of Texture Studies 46 (2015): 429–439, 10.1111/jtxs.12142.

[29] T. Aktar , J. Chen , R. Ettelaie , and M. Holmes , “Touch Sensation and Viscosity Discrimination,” Journal of Sensory Studies 30 (2015): 98–107, 10.1111/joss.12141.

[30] J. J. Gibson , “Observations on Active Touch,” Psychological Review 69 (1962): 477–491, 10.1037/h0046962. 13947730

[31] S. J. Bolanowski , R. T. Verrillo , and F. McGlone , “Passive, Active and Intra‐Active (Self) Touch,” Somatosensory & Motor Research 16 (1999): 304–311, 10.1080/08990229970375. 10632028

[32] C. Simões‐Franklin , T. A. Whitaker , and F. N. Newell , “Active and Passive Touch Differentially Activate Somatosensory Cortex in Texture Perception,” Human Brain Mapping 32 (2011): 1067–1080, 10.1002/hbm.21091. PMC6870332 20669167

[33] H. Maezawa , Y. Hirai , H. Shiraishi , and M. Funahashi , “Somatosensory Evoked Magnetic Fields Following Tongue and Hard Palate Stimulation on the Preferred Chewing Side,” Journal of the Neurological Sciences 347 (2014): 288–294, 10.1016/j.jns.2014.10.025. 25455302

[34] M. F. Folstein , S. E. Folstein , and P. R. McHugh , “‘Mini‐Mental State’. A Practical Method for Grading the Cognitive State of Patients for the Clinician,” Journal of Psychiatric Research 12 (1975): 189–198, 10.1016/0022-3956(75)90026-6. 1202204

[35] P. C. Belafsky , D. A. Mouadeb , C. J. Rees , et al., “Validity and Reliability of the Eating Assessment Tool (EAT‐10),” Annals of Otology, Rhinology, and Laryngology 117 (2008): 919–924, 10.1177/000348940811701210. 19140539

[36] S. P. McKee , S. A. Klein , and D. Y. Teller , “Statistical Properties of Forced‐Choice Psychometric Functions: Implications of Probit Analysis,” Perception & Psychophysics 37 (1985): 286–298, 10.3758/BF03211350. 4034345

[37] R. Ulrich and J. Miller , “Threshold Estimation in Two‐Alternative Forced‐Choice (2AFC) Tasks: The Spearman‐Kärber Method,” Perception & Psychophysics 66 (2004): 517–533, 10.3758/BF03194898. 15283075

[38] M. Ohta , Y. Imamura , N. Chebib , et al., “Oral Function and Nutritional Status in Non‐Acute Hospitalised Elders,” Gerodontology 39 (2022): 74–82, 10.1111/ger.12612. PMC9299802 34913521

[39] H. Akemi , O. Yoshiaki , and M. Yoshuharu , “Examination of Bolus Transport in the Oral Stage of Swallowing Using an Ultrasound Duplex—Doppler Analysis Standard,” Japanese Journal of Dysphagia Rehabilitation 6 (2002): 9–18, 10.32136/jsdr.6.1_9.

[40] M. Trulsson and G. K. Essick , “Low‐Threshold Mechanoreceptive Afferents in the Human Lingual Nerve,” Journal of Neurophysiology 77 (1997): 737–748, 10.1152/jn.1997.77.2.737. 9065846

[41] M. Trulsson and G. K. Essick , “Sensations Evoked by Microstimulation of Single Mechanoreceptive Afferents Innervating the Human Face and Mouth,” Journal of Neurophysiology 103 (2010): 1741–1747, 10.1152/jn.01146.2009. 20130037

[42] Y. Ito , E. Kosei , T. Yasue , Y. Satoshi , and H. Yoshinori , “Association Between Texture Sensation in Tongue‐Hard Palate and Morphometric Characteristics of Lingual Papillae: A Pilot Study [In Japanese],” Journal of the Japanese Society of Stomatognathic Function 23 (2016): 85–95, 10.7144/sgf.23.85.

[43] K. Kobayashi , M. Kumakura , K. Yoshimura , and J. Shindo , “Stereo‐Structural Study of the Lingual Papillae and Their Connective Tissue Cores in Relation to Ageing Changes in the Human Tongue,” Italian Journal of Anatomy and Embryology 106 (2001): 305–311. 11729970

[44] T. Yoko , K. Junichi , K. Junpei , and K. Yasuaki , “Relationship Between Lingual Papilla Surface Roughness and Oral Condition in Elderly Individuals,” Open Journal of Stomatology 11 (2021): 77–90, 10.4236/ojst.2021.112007.

[45] R. G. Bangcuyo and C. T. Simons , “Lingual Tactile Sensitivity: Effect of Age Group, Sex, and Fungiform Papillae Density,” Experimental Brain Research 235 (2017): 2679–2688, 10.1007/s00221-017-5003-7. 28589232

[46] A. J. Johnson , A. T. Wilson , C. L. Nodarse , et al., “Age Differences in Multimodal Quantitative Sensory Testing and Associations With Brain Volume,” Innovation in Aging 5 (2021): igab033, 10.1093/geroni/igab033. PMC8489433 34616958

[47] S. McIntyre , S. S. Nagi , F. McGlone , and H. Olausson , “The Effects of Ageing on Tactile Function in Humans,” Neuroscience 464 (2021): 53–58, 10.1016/j.neuroscience.2021.02.015. 33607227

[48] Y. Moayedi , S. Michlig , M. Park , A. Koch , and E. A. Lumpkin , “Somatosensory Innervation of Healthy Human Oral Tissues,” Journal of Comparative Neurology 529 (2021): 3046–3061, 10.1002/cne.25148. PMC10052750 33786834

[49] J. Scheibert , S. Leurent , A. Prevost , and G. Debrégeas , “The Role of Fingerprints in the Coding of Tactile Information Probed With a Biomimetic Sensor,” Science 323 (2009): 1503–1506, 10.1126/science.1166467. 19179493

